# Production and Composition of Group B Streptococcal Membrane Vesicles Vary Across Diverse Lineages

**DOI:** 10.3389/fmicb.2021.770499

**Published:** 2021-11-22

**Authors:** Cole R. McCutcheon, Macy E. Pell, Jennifer A. Gaddy, David M. Aronoff, Margaret G. Petroff, Shannon D. Manning

**Affiliations:** ^1^Department of Microbiology and Molecular Genetics, Michigan State University, East Lansing, MI, United States; ^2^Division of Infectious Disease, Department of Medicine, Vanderbilt University Medical Center, Nashville, TN, United States; ^3^Department of Pathology, Microbiology, and Immunology, Vanderbilt University Medical Center, Nashville, TN, United States; ^4^Tennessee Valley Healthcare System, Department of Veterans Affairs, Nashville, TN, United States; ^5^Department of Obstetrics and Gynecology, Vanderbilt University Medical Center, Nashville, TN, United States; ^6^Department of Pathobiology and Diagnostic Investigation, Michigan State University, East Lansing, MI, United States

**Keywords:** *Streptococcus agalactiae*, membrane vesicles, virulence, pathogenesis, proteomics, group B *Streptococcus*

## Abstract

Although the neonatal and fetal pathogen Group B *Streptococcus* (GBS) asymptomatically colonizes the vaginal tract of ∼30% of pregnant women, only a fraction of their offspring develops invasive disease. We and others have postulated that these dimorphic clinical phenotypes are driven by strain variability; however, the bacterial factors that promote these divergent clinical phenotypes remain unclear. It was previously shown that GBS produces membrane vesicles (MVs) that contain active virulence factors capable of inducing adverse pregnancy outcomes. Because the relationship between strain variation and vesicle composition or production is unknown, we sought to quantify MV production and examine the protein composition, using label-free proteomics on MVs produced by diverse clinical GBS strains representing three phylogenetically distinct lineages. We found that MV production varied across strains, with certain strains displaying nearly twofold increases in production relative to others. Hierarchical clustering and principal component analysis of the proteomes revealed that MV composition is lineage-dependent but independent of clinical phenotype. Multiple proteins that contribute to virulence or immunomodulation, including hyaluronidase, C5a peptidase, and sialidases, were differentially abundant in MVs, and were partially responsible for this divergence. Together, these data indicate that production and composition of GBS MVs vary in a strain-dependent manner, suggesting that MVs have lineage-specific functions relating to virulence. Such differences may contribute to variation in clinical phenotypes observed among individuals infected with GBS strains representing distinct lineages.

## Introduction

Group B *Streptococcus* (GBS) is an opportunistic pathogen that asymptomatically colonizes ∼30% of women either vaginally or rectally (Verani et al., 2010). In individuals with a compromised or altered immune state, including pregnant women, neonates, the elderly, and people living with diabetes mellitus, GBS can cause severe infections (Verani et al., 2010). Presentation of disease is variable between individuals: in elderly patients and neonates, GBS infection typically presents as septicemia, whereas in pregnant women it more commonly causes chorioamnionitis, preterm birth, or stillbirth (Doran and Nizet, 2004; Edwards and Baker, 2005).

Despite the high prevalence of GBS colonization during pregnancy, only a fraction of babies born to colonized mothers develops an infection. In the United States pregnant individuals colonized with GBS are given antibiotics to reduce the risk of neonatal GBS infection, but even without such prophylaxis most neonates born to GBS-colonized mothers remain infection-free (Aronoff and Blaser, 2020). The factors that determine whether a neonate develops GBS sepsis or not are incompletely understood, but evidence implicates bacterial strain variation as a key factor. For example, certain polysaccharide capsular serotypes of GBS are much more common at causing perinatal infections than others (Bianchi-Jassir et al., 2020).

Application of multilocus sequence typing (MLST) has also demonstrated that GBS isolates comprise multiple sequence types (STs) that are differentially correlated with disease outcomes (Jones et al., 2003). While ST-12 strains have been associated with asymptomatic colonization (Manning et al., 2008), ST-1 and ST-17 strains have been linked to invasive disease in adults and neonates, respectively (Jones et al., 2003; Poyart et al., 2008; Manning et al., 2009; Flores et al., 2015). Moreover, our group has previously shown that different STs interact variably with host cells. ST-17 strains, for instance, had an enhanced ability to attach to gestational tissues, elicited stronger proinflammatory responses, and could persist longer inside macrophages than other STs (Korir et al., 2014, 2017; Flaherty et al., 2019). Conversely, ST-12 strains were found to display increased tolerance to ampicillin relative to ST-17 strains (Korir et al., 2017), highlighting the divergence of these lineages and variation in the ability to withstand different stressors. The mechanisms underlying these strain-dependent differences, however, are poorly understood.

Many bacteria produce membrane vesicles (MVs) of varying sizes (20–500 nm) containing toxins and other virulence factors that can modulate immune responses and influence pathogenesis (Brown et al., 2015). In addition, GBS can produce MVs that have been implicated in infection risk, though this remains an area in need of more research (Surve et al., 2016; Armistead et al., 2021). While the exact role of GBS MVs in pathogenesis is not clear, intra-amniotic injection of GBS MVs produced by an invasive ST-7 strain induced preterm birth and intrauterine fetal death in mice (Surve et al., 2016). GBS MVs were also found to contain active virulence factors that could weaken murine gestational membranes, stimulate immune cell recruitment, and lyse host cells (Surve et al., 2016; Armistead et al., 2021). Hence, an important, unanswered question is whether MVs derived from strains belonging to distinct phylogenetic lineages and clinical sources vary in composition and pathogenic potential.

In this study, we sought to compare the quantity and protein composition of MVs produced by genetically distinct GBS strains and evaluate the relationships between proteomic profiles, strain characteristics, and clinical presentation. To accomplish these goals, we isolated MVs from six clinical strains representing three phylogenetic lineages (ST-1, ST-12, and ST-17), and used label-free proteomics to define the protein composition. Using this approach, we report that MV production and composition vary in a strain- and ST-dependent manner, highlighting the importance of strain diversity on pathogenicity and virulence.

## Materials and Methods

### Bacterial Strains, Growth Conditions, and Growth Curves

Six GBS strains, GB00020, GB00037, GB00112, GB00411, GB00653, and GB01455, were isolated as described (Davies et al., 2001; Spaetgens et al., 2002); the strain names have been abbreviated for clarity. The six strains were selected for inclusion based on the isolation source and epidemiological data as well as the ST and capsular (cps) serotype designations. Three strains, GB37, GB411, and GB1455, were classified as “invasive” because they were isolated from septic neonates with early onset GBS disease or were cultured from a stillbirth (GB1455) (Davies et al., 2001). The three “colonizing” isolates, GB20, GB112, and GB653, were isolated from vaginal/rectal swabs from mothers before or after childbirth (Spaetgens et al., 2002). All six strains were previously characterized by MLST and cps typing (Manning et al., 2008, 2009) and represent the following common ST/serotype combinations: ST-1, cpsV (GB20, GB37); ST-12, cpsII (GB653, GB1455); and ST-17, cpsIII (GB112, GB411). One colonizing and one invasive strain were included in each of the ST/cps groups.

Because we had no prior knowledge of MV production across lineages, these strains were selected based on molecular data as well as epidemiological and clinical associations described previously. The ST-17 strains, for instance, have consistently been associated with invasive neonatal disease (Jones et al., 2003; Manning et al., 2008; Poyart et al., 2008) and were more likely to persist in mothers following childbirth and intrapartum antibiotic prophylaxis (IAP) (Manning et al., 2008). ST-12 strains, however, were more common during pregnancy and more readily lost following IAP (Manning et al., 2008, 2009). Although ST-1 strains have been linked to invasive disease in adults (Flores et al., 2015), they were more commonly recovered from women during pregnancy than neonates in our studies (Manning et al., 2008, 2009). It is also notable that the ST-1 neonatal GB37 strain has unique traits in that it is non-pigmented and non-hemolytic (Singh et al., 2016). This diverse set of strains with varying characteristics and epidemiological associations was chosen to maximize our ability to detect differences in MV production across strains.

Strains were cultured using Todd-Hewitt Broth (THB) or Todd-Hewitt Agar (THA) (BD Diagnostics, Franklin Lakes, New Jersey, United States) overnight at 37°C with 5% CO_2_. For enumeration of colony forming units (CFUs), bacteria were serially diluted in Phosphate Buffered Saline (PBS) and plated onto THA. Plates with 20–200 colonies were counted and the number of colonies per mL was determined. Growth curves were performed by diluting overnight THB cultures 1:50 into fresh THB. Cultures were grown for 6 h with OD_600_ measurements taken hourly. Growth curves were performed in triplicate for each isolate.

### Membrane Vesicle Isolation and Purification

The isolation and purification of MVs was performed as described (Chutkan et al., 2013; Klimentová and Stulík, 2015; Surve et al., 2016; Nguyen et al., 2021), with some modifications. Briefly, overnight THB cultures were diluted 1:50 into fresh broth and grown to late logarithmic phase (optical density at 600 nm, OD_600_ = 0.9 ± 0.05). Aliquots of culture were serially diluted and plated on THA for bacterial enumeration. Cultures were centrifuged at 2,000 × g for 20 min at 4°C. Supernatants were collected and re-centrifuged at 8,500 × g for 15 min at 4°C, followed by filtration through a 0.22 μm filter and concentration using Amicon Ultra-15 centrifugal filters (10 kDa cutoff) (Millipore Sigma, Burlington, MA, United States). Concentrated supernatants were subjected to ultracentrifugation for 2 h at 150,000 × g at 4°C. For quantification, pellets were washed by resuspending in PBS, re-pelleting at 150,000 × g at 4°C, and resuspending in PBS; pellets were stored at −80°C until usage.

For proteomics, pellets were resuspended in PBS and purified using qEV Single size exclusion columns (IZON Science, Christchurch, New Zealand) per the manufacturer’s instructions. MV fractions were collected and re-concentrated using the Amicon Ultra-4 centrifugal filters (10 kDa cutoff) (MilliporeSigma, Burlington, Massachusetts, United States) and brought to a final volume of 100 μL in PBS. To preserve the integrity of vesicle proteins, ProBlock Gold Bacterial Protease Inhibitor Cocktail (GoldBio, St. Louis, Missouri, United States) was added. MVs were stored at −80°C until usage.

### Electron Microscopy

To visualize GBS and the MVs associated with each strain, scanning electron microscopy (SEM) was performed on bacterial cultures grown to stationary phase in THB. Culture aliquots were fixed in equal volumes of 4% glutaraldehyde in 0.1 M phosphate buffered saline (pH 7.4), placed on poly-L-lysine coated 12 mm coverslips, and incubated for 5 min. The coverslips were washed with water and dehydrated through increasing concentrations of ethanol (25, 50, 75, and 95%) for 5 min in each followed by three 5-min changes in 100% ethanol. Samples were dried in a Leica Microsystems (model EM CPD300) critical point drier using liquid carbon dioxide as the transitional field. Lastly, samples were mounted on aluminum stubs using epoxy glue (System Three Quick Cure 5, System Three Resins, Inc., Lacey, Washington, United States) and coated with osmium (∼10 mm thickness) using a NEOC-AT osmium coater (Meiwafosis Co., Ltd., Tokyo, Japan). Imaging was performed using a JEOL 7500F scanning electron microscope.

To evaluate MV morphology and purity without contaminating extracellular components, transmission electron microscopy (TEM) was performed on purified vesicles as described (Nguyen et al., 2021). MVs were fixed in 4% paraformaldehyde, loaded onto formvar-carbon coated grids, and counterstained with 2.5% glutaraldehyde and 0.1% uranyl acetate in PBS. Samples were imaged using a JEOL 1400 Flash transmission electron microscope. During proteomics experiments, preparations with high concentration of MVs and minimal extravesicular contamination were included for downstream analyses. Each proteomics preparation was imaged with TEM prior to analysis to confirm the presence of spherical MVs.

### Quantification of Vesicle Production

Nanoparticle tracking analysis was performed to quantify MVs produced by each strain (*n* = 8–9 replicates per strain) using a NanoSight NS300 (Malvern Panalytical Westborough, MA, United States) equipped with an automated syringe sampler as described previously (Nguyen et al., 2019, 2021). For each sample, MVs were diluted in phosphate buffered saline (1:100--1:1,000) and injected with a flow rate of 50. Once loaded, five 20-s videos were recorded at a screen gain of 1 and camera level of 13. After capture, videos were analyzed at a screen gain of 10 and a detection threshold of 4 and data were subsequently exported to a CSV file for analysis using the R package tidyNano^1^ (Nguyen et al., 2019). Total MV counts were normalized by dividing by the colony forming units (CFUs) of each original bacterial culture following growth to an OD_600_ of 0.9 ± 0.05. Differences in MV quantities were assessed using the Kruskal Wallis test followed by a *post hoc* Dunn’s Test with a Benjamini-Hochberg correction. Outliers were identified by multiplying the interquartile range by 1.5, which was used to extend the upper and lower quartiles.

### Proteomics and Genomics

Proteomic LC-MS/MS analysis of MVs was performed in duplicate or triplicate by the Proteomics Core at the Michigan State University Research Technology Support Facility (RTSF). Protein concentrations of purified MVs were determined using the Pierce Bicinchoninic Acid Assay (Thermo Fisher Scientific, Waltham, Massachusetts) supplemented with 2% SDS in water to reduce the background signal from excess lipids contained within the vesicles. MVs (1.5 μg) were concentrated into a single band in a 4–20% Tris-Glycine SDS-PAGE gel (BioRad, Hercules, CA) that was fixed and stained using colloidal Coomassie blue (Dyballa and Metzger, 2009).

Protein bands were excised from the gels and stored in 5% acetic acid at 4°C. Prior to analysis, in-gel trypsin digest and peptide extraction were performed. Briefly, gel bands were dehydrated twice using 100% acetonitrile and incubated with 10 mM dithiothreitol in 100 mM ammonium bicarbonate (pH∼8.0) at 56°C for 45 min. Bands were incubated in the dark with 50 mM iodoacetamide in 100 mM ammonium bicarbonate for 20 min followed by another dehydration. Sequencing grade modified trypsin (0.01 μg/μL in 50 mM ammonium bicarbonate) was added to each gel band and incubated at 37°C overnight. Peptides extracted by bath sonication (in 60% acetonitrile, 1% trichloroacetic acid solution) were vacuum dried and re-suspended (in 2% acetonitrile/0.1% trifluoroacetic) prior to separation using a Thermo ACCLAIM C18 trapping column. Peptides were sprayed onto a Thermo Fisher Q-Exactive HFX mass spectrometer for 90 min; the top 30 ions per survey were analyzed further using high energy induced dissociation. MS/MS spectra were converted into peak lists using Mascot Distiller v2.7.0 and searched against a SwissProt database containing all GBS sequences available through the National Center for Biotechnology Information (NCBI; accessed 2/08/2019). Contaminants were identified and removed using Mascot searching algorithm v2.7.0, while protein identities were validated using Scaffold v4.11.1. Raw proteomic data was submitted to the MassIVE database and can be accessed via ftp://MSV000087985@massive.ucsd.edu or at doi: 10.25345/C5RC1H.

Whole-genome sequencing was performed previously on GB00020 (Parker et al., 2017) and GB00037 (Singh et al., 2016). These genomes were examined more comprehensively to confirm the presence of specific genes found to be absent in the proteomics analysis. Raw reads were trimmed using Trimmomatic 0.39 (Bolger et al., 2014) followed by an assessment of sequence quality using FastQC (Barbraham Bioinformatics*). De novo* genome assembly was performed on high-quality paired-end reads using SPAdes 3.13.1 (Prjibelski et al., 2020). Assembly quality was assessed using QUAST 5.0.2. Protein sequences were downloaded from GenBank and aligned to assembled contigs using tblastn. Proteins with 90% identity or higher were considered present.

### Data Analysis

To compare MV proteins between strains, proteomic data from all strains were compiled and normalized for inter-experimental variability using Scaffold. Only proteins with a minimum of two identified peptides falling above a 1% false discovery rate and 95% protein threshold, were considered for downstream analysis. Proteins identified as contaminants (via the Mascot searching algorithm v 2.6.0) were removed, whereas proteins identified in both replicates for at least one strain were classified as MV-associated. Subcellular localization analysis was performed using pSORTdb^2^ with protein localization data for GBS strain 2603VR (downloaded from pSORTdb on 3/6/2021). Data visualization and statistical analyses were performed using R version 4.1.0.^3^ Principle component analysis (PCA) was performed and visualized using the prcomp and fviz_pca functions, respectively. Hierarchical clustering was performed using the pheatmap function and clustered using Euclidean distances. Shapiro tests were used to determine whether data followed a normal distribution and Student *t*-test (two-sided) or Kruskal-Wallis one-way analysis of variance (ANOVA), in combination with the Dunn’s *post hoc* test, were utilized to test for differences between groups. Multiple hypothesis testing was corrected using Benjamini-Hochberg or Bonferroni correction when necessary.

## Results

### Membrane Vesicles Are Produced by Different Group B *Streptococcus* Strains Representing Common Sequence Types

Prior to MV isolation, each strain was monitored for growth, which did not differ significantly throughout the logarithmic phase (Supplementary Figure 1). Although a slight decrease in OD_600_ was observed for GB1455 in early stationary phase, all strains reached late logarithmic/early stationary phase at an OD_600_ of 0.9. In addition, all strains displayed a similar length of each growth phase, suggesting minimal differences in growth dynamics.

**FIGURE 1:**
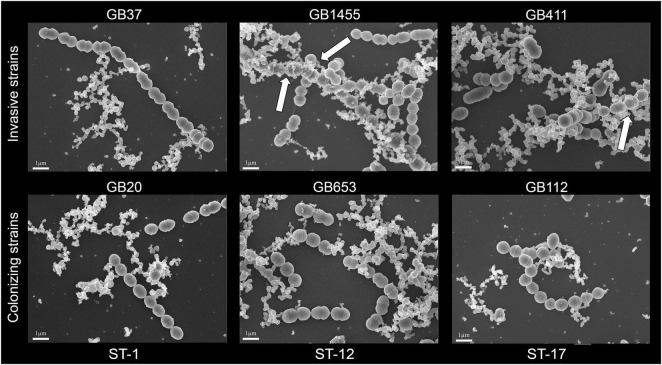
Scanning electron microscopy (SEM) of membrane vesicles (MVs) from overnight cultures of six group B streptococcal strains, which were visualized by SEM at 10,000× magnification following growth to stationary phase. White arrows show MVs that are closely associated with bacterial cells. A minimum of 2 replicates per strain were examined and the SEM scale bars represent 1 μm length.

**FIGURE 2:**
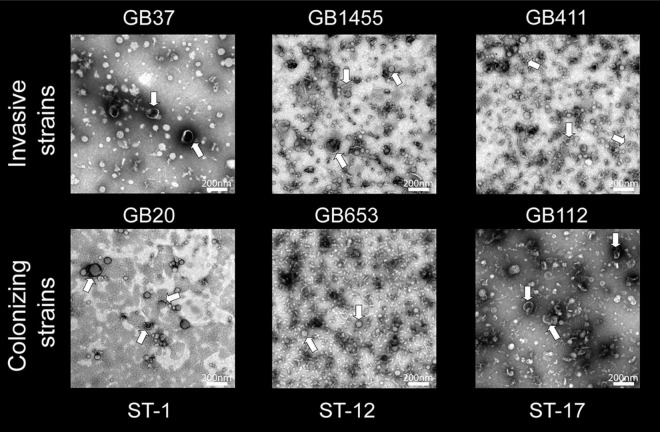
Transmission electron microscopy (TEM) of membrane vesicles (MVs) isolated from the six group B streptococcal strains. An abundance of spherical structures with a bright membrane bilayer and slightly electron dense interior was observed ranging in size between ∼50 and 100 nm. The MV population was isolated from each strain following late logarithmic growth. MVs were purified using ultracentrifugation and size exclusion chromatography (2–3 replicates per strain). TEM images were taken at a magnification of 20,000×; the scale bars indicate a length of 200 nm.

To determine whether each of the six GBS strains could produce MVs, we first used SEM to examine bacterial cultures grown overnight to stationary phase (Figure 1). Visualization using SEM revealed abundant production of MVs by all six strains and showed that some MVs were closely associated with bacterial cells as was described in prior studies (Brown et al., 2015). Because these overnight cultures likely contain cellular debris as well as MVs, further confirmation was necessary to rule out extra-vesicular contamination. To limit the possibility of detecting debris in the MV preparations, we grew each of the six strains to late logarithmic phase at an OD_600_ of 0.9 ± 0.05 prior to MV isolation and purification. Imaging by TEM revealed that MVs were produced by all six strains. On average, they ranged in diameter between ∼50 and 100 nm and displayed a spherical morphology with a lipid bilayer and slightly electron dense interior (noted by arrows in Figure 2). The MVs appeared similar to other bacterial-derived MVs described in the literature (Brown et al., 2015) and for GBS strain A909 (Surve et al., 2016).

### The Level of Membrane Vesicle Production Differs Across Group B *Streptococcus* Strains

**FIGURE 3:**
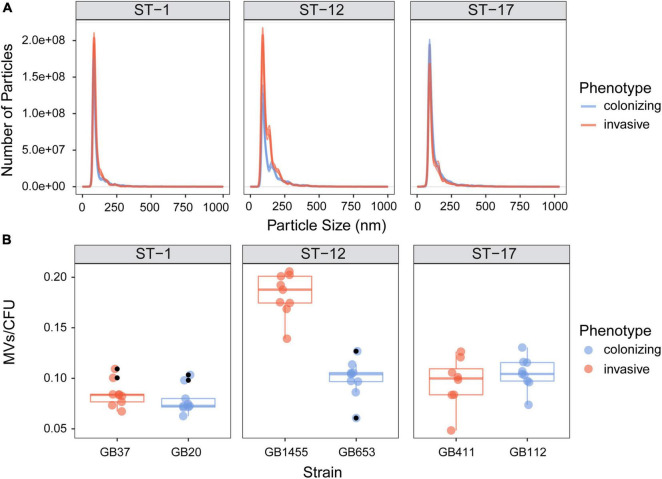
Quantitative assessment of membrane vesicle (MV) production across strains. MVs were isolated by differential centrifugation and quantified using NanoSight analysis. The vesicle **(A)** size distribution and **(B)** number per bacterial colony forming units (CFUs) are shown for the invasive and colonizing strains by sequence type (ST). For panel A, the lines represent the mean, while the shading surrounding the mean is the standard error of the mean. For panel B, total MV counts were normalized to the number of CFUs in the original bacterial cultures. The horizontal lines within each box show the median across 8–9 biological replicates (indicated by colored dots). The black dots represent outliers identified by multiplying the interquartile range by 1.5, which was used to extend the upper and lower quartiles. Outliers were observed for three of the six strains and were excluded prior to statistical analysis. GB1455 produced significantly more MVs than all other strains (*p* < 0.05) and GB20 produced significantly fewer MVs relative to all other strains (*p* < 0.05) except GB37.

Because electron microscopy suggested differences in MV production, we used NanoSight analysis to quantify MV size and production. MVs from each of the six strains displayed a uniform size distribution, ranging between 100 and 200 nm (Figure 3A). Similar size distributions were also observed by ST. For MV quantification, total MV counts were normalized to the number of CFUs in the original bacterial cultures. Among the six strains, the average number of MVs/CFU was 0.108 with a range of 0.048–0.206 MVs/CFU; however, there was considerable variation between strains (Figure 3B). Although no difference in MV quantity was observed in colonizing vs. invasive strains belonging to ST-1 or ST-17, the ST-1 strains produced significantly fewer MVs relative to the ST-17 strains (Supplementary Figure 2; *p* < 0.0001). While the colonizing ST-12 (cpsII) GB653 strain produced similar vesicle quantities as the two ST-17 (cpsIII) strains, the invasive ST-12 (cpsII) isolate, GB1455, produced significantly more MVs than all other strains examined (*p* < 0.05). By contrast, the colonizing ST-1 (cpsV) isolate, GB20, produced significantly fewer MVs compared to the strains representing all other STs (*p* < 0.05) except for the other ST-1 (GB37) strain (*p* = 0.55).

### Variation in Membrane Vesicle Protein Abundance and Identification of a Shared Proteome

**FIGURE 4:**
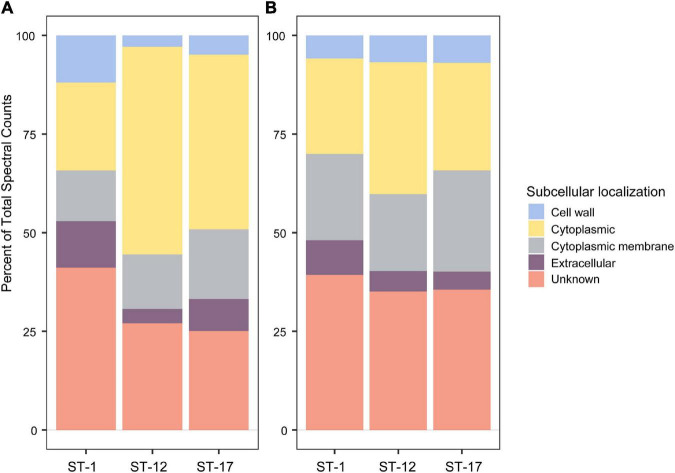
Subcellular localization analysis of membrane vesicle (MV) proteomes. The subcellular localization of **(A)** all 643 MV proteins identified, and **(B)** a subset of 62 shared MV proteins identified using a pSORTdb database for published *Streptococcus agalactiae* sequences (accessed 3/3/21). Percentages were determined from mean spectral counts for a given sequence type (ST).

Proteomics of purified MVs identified 643 total proteins among the six isolates with an average of 458 proteins per strain and range of 239–614 proteins per strain (Supplementary Table 1). Of note, the number of unique proteins varied by strain. MVs from ST-1 strains, for instance, had fewer unique proteins relative to the other STs with an average of 281 proteins compared to 601 and 493 for the ST-12 and ST-17 strains, respectively. Regardless of ST, however, pSORTdb predicted numerous proteins to be membrane (12–17%) and cell wall (2–11%) localized, while 22–52% were predicted to be localized in the cytoplasm (Figure 4A). Although many proteins had a predicted subcellular localization, a large proportion of proteins had unidentified or unpredicted subcellular localization.

Among the total proteins detected, 62 were found in all biological replicates for the six strains (Supplementary Table 2). These proteins did not vary in spectral abundance between STs and represent the shared MV proteome. Of these 62 proteins, 11 were highly abundant with a mean spectral count greater than 50 (Supplementary Table 3). Putative, uncharacterized transporters constituted many of these shared proteins, accounting for 39–44% of membrane protein spectral counts. In addition, 19–25% of spectral counts were predicted to have a membrane associated subcellular localization (Figure 4B).

**FIGURE 5:**
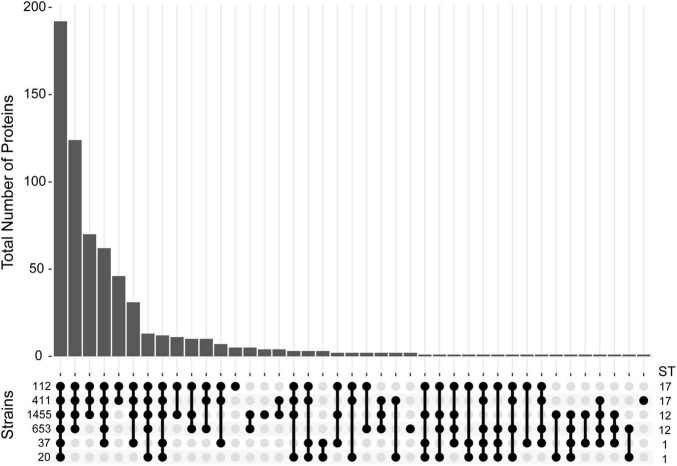
Distribution of proteins detected in membrane vesicles (MVs) among six strains. An Upset plot was generated to show the distribution of all 643 proteins detected across the six GBS strains examined. The *y*-axis indicates the total number of proteins detected for a given set of strains. Protein presence is defined as having a non-zero spectral count for a given protein in at least one biological replicate for a specific strain. The matrix at the base of the plot shows the strains ordered vertically by sequence type (ST) with filled bubbles indicating which strains are positive for the number of proteins detected, and overlaid bars representing number of shared proteins.

In other species, studies have demonstrated that MV composition can vary across strains, which could confer strain specific functionality (Jeon et al., 2016; Tandberg et al., 2016). Therefore, we sought to determine how many of these proteins were strain-specific or shared among the six strains examined. Of all 643 proteins detected, 192 (29.9%) were detected in at least one biological replicate for all six strains regardless of the clinical phenotype or ST (Figure 5). This analysis enhanced our certainty that a protein was present in a given strain, while permitting us to compare its abundance across strains even if it was not detected. Notably, 124 (19.3%) proteins were shared by the four ST-12 and ST-17 strains but were absent in the ST-1 strains, suggesting that the ST-1 MVs have a unique protein composition. To determine whether these compositional differences were due to genome divergence, analysis of whole-genome sequencing data revealed that 122 of the 124 corresponding protein genes were present in the ST-1 genomes. Interestingly, the two proteins absent from these genomes were ARC24477.1 and ARC24478.1 encoding a CHAP-domain containing protein and an abortive phage resistance protein, respectively, both of which are located within a putative phage. Although a minor proportion of proteins were ST- or strain specific, none were shared by all invasive or all colonizing strains.

### Compositional Protein Profiles Differ Across Group B *Streptococcus* Membrane Vesicles

**FIGURE 6:**
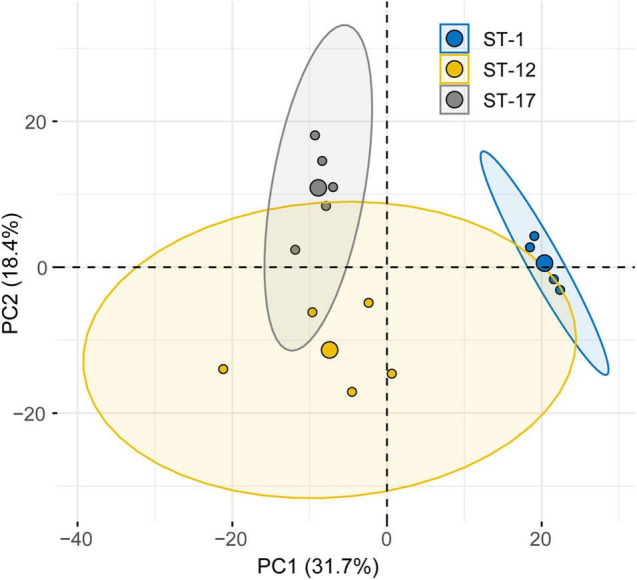
Principal component analysis (PCA) reveals lineage-specific clustering of membrane vesicle (MV) proteomes. PCA of the MV proteomes produced by six strains stratified by sequence type (ST). The large central dot of each ellipse represents the mean point of the corresponding 95% confidence ellipse, while the smaller points represent individual proteomic samples. Confidence ellipses comprise 95% of the samples based on the underlying distribution. Axes percentages represent the amount of variation accounted for by each principal component (PC).

Given that differences in protein abundance were observed, we next considered the relationship between protein composition and strain characteristics. Rather than differential protein abundance analysis, we assessed whole proteome composition using PCA (Figure 6). This method takes into consideration the spectral abundance of all proteins simultaneously, giving a more thorough evaluation of population level changes in composition. In our analysis we found that the first two principal components accounted for a high proportion of the total variation (50.1%). Although the protein composition of MVs from invasive and colonizing strains overlapped, it was segregated by ST. Some overlap, however, was observed between the ST-12 confidence ellipse and those for other STs. No overlap was seen between the ST-1 and ST-17 strains, highlighting their distinct proteomes. This distinct clustering was not observed when the relationship between protein composition and clinical phenotype was analyzed (Supplementary Figure 3). Specifically, invasive and colonizing samples displayed a high degree of overlap with little to no separation of their respective confidence ellipses.

**FIGURE 7:**
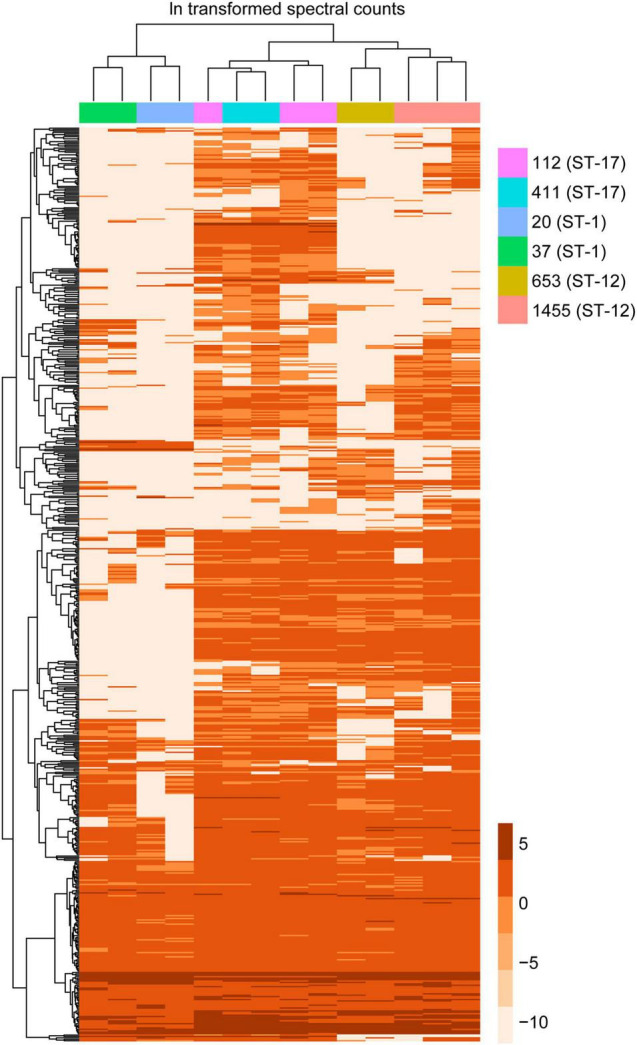
Hierarchical clustering of membrane vesicle (MV) proteomes shows sequence type (ST) specific clustering. A heatmap was generated using hierarchical clustering with the pheatmap function in R, which uses Euclidean distance to cluster rows and columns with similar profiles. Individual rows represent a single accession number for an identified protein, with the color gradient of individual boxes corresponding to the natural log (ln) transformation of spectral counts for a given protein of interest. Columns represent a single proteomic sample, which are color coded by strain.

To confirm the PCA results, we then applied a hierarchical clustering algorithm to our dataset, which utilizes a different statistical assessment to evaluate the relationship between MV composition and various strain characteristics. Indeed, hierarchical clustering of the protein data further demonstrated that MVs from strains belonging to the same ST had similar protein profiles, forming distinct clusters by ST regardless of the clinical phenotype (Figure 7). For instance, proteins from the ST-12 and ST-17 strains formed a distinct branch in the phylogeny that was separate from the ST-1 proteins, thereby indicating that their protein composition was more similar to each other than to ST-1 strains. This observation supports the PCA, showing a higher degree of overlap between ST-12 and ST-17 strains compared to ST-1 strains. Nonetheless, ST-12 and ST-17 strains were still distinguishable, with distinct nodes forming based on protein composition, indicating their divergent composition. This analysis provided additional confirmation that ST-1 strains lacked several proteins that were highly abundant in both the ST-12 and ST-17 strains. To a lesser degree than the ST-1 MVs, several highly abundant proteins found among the ST-17 strains were also absent in the ST-12 strains.

### Differential Abundance of Key Virulence Factors in Membrane Vesicles From Distinct Group B *Streptococcus* Strains

**FIGURE 8:**
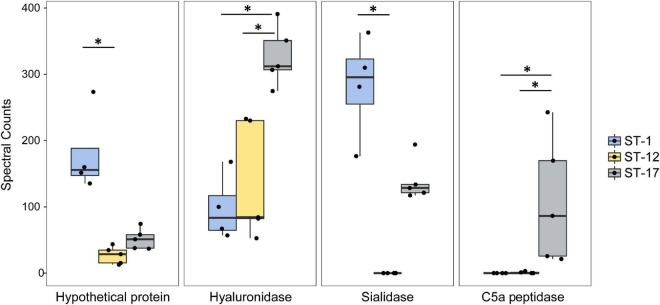
Highly abundant proteins are present at variable levels in group B streptococcal membrane vesicles (MVs). The spectral counts of specific proteins were plotted after stratifying by the sequence type (ST). The median spectral count associated with each ST is represented within each box. The black dots represent a single biological replicate for a given strain. Statistical comparison was performed using a Kruskal Wallis test. Multiple pairwise comparisons were then made using the pairw.kw function in R, which uses a conservative Bonferroni correction method to correct for multiple hypothesis testing. Comparisons with *p*-values < 0.05 are denoted with an asterisk.

To determine which proteins contributed most to the segregation observed in the PCA as well as the hierarchical clustering analysis, we more thoroughly examined the 335 proteins that were significantly enriched in at least one ST (Supplementary Table 4). Notably, several purported virulence factors including the C5a peptidase, hyaluronidase, and sialidase were highly enriched in a ST-dependent manner (Figure 8). Both the hyaluronidase and C5a peptidase were significantly more abundant in the two ST-17 strains compared to the ST-1 and ST-12 strains, whereas the sialidase was detected at significantly higher levels in ST-1 vs. ST-12 strains.

Several proteins of unknown function were also among the most highly abundant and differentially enriched proteins detected. One hypothetical protein, for instance, was significantly more abundant in the ST-1 strains relative to strains representing the other two lineages (Figure 8). Similarly, another hypothetical protein was more abundant in the ST-12 strains (Supplementary Figure 4); however, considerable variation was observed across replicates. Numerous phage-associated proteins including a holin and capsid protein, were also detected and found to be more abundant in the ST-17 strains along with several proteins associated with cell division (Supplementary Figure 5). For example, the average abundance of cell division proteins FtsE, FtsQ, FtsZ, and FtsY, was significantly greater in the two ST-17 strains compared to those from other lineages. Differences in proteins linked to cell wall modification such as penicillin-binding proteins and capsule biosynthesis proteins, were also detected (Supplementary Figure 6).

## Discussion

Current knowledge regarding GBS derived MVs is restricted to one clinical strain (Surve et al., 2016; Armistead et al., 2021) and hence, we sought to examine MV production and composition in a set of clinical strains with different traits. While no clear association was observed between clinical phenotype and the production or composition of MVs, we have demonstrated that the GBS MV proteome is ST-dependent. The same was observed for MV production, though some variation was noted between strains of the same ST. Together, these data indicate that GBS MVs have strain-dependent functions that could impact survival in hosts, immunomodulation, and virulence.

This study expands our current knowledge of GBS MVs by highlighting their potential impact on virulence. Specifically, we demonstrated that GBS MVs have a high abundance of immunomodulatory virulence factors including C5a peptidase, hyaluronidase, and sialidase (Cheng et al., 2002; Kolar et al., 2015; Yamaguchi et al., 2016). The bifunctional C5a peptidase has been shown to interact with fibronectin and degrade the proinflammatory complement component (C5a) while simultaneously promoting bacterial invasion into host cells (Cheng et al., 2002; Kolar et al., 2015). MVs from both ST-17 (cpsIII) strains examined herein contained high levels of C5a peptidase, whereas ST-1 and ST-12 strains lacked this protein. Intriguingly, ST-17 strains were previously shown to possess distinct virulence gene profiles as well as unique alleles of *scpB* encoding the C5a peptidase (Brochet et al., 2006; Springman et al., 2009), suggesting that ST-17 strains may be primed to cause invasive infections. This suggestion is in line with epidemiological data showing that ST-17 strains are important for invasive disease in adults and neonates (Jones et al., 2003; Manning et al., 2009; Flores et al., 2015) as well as mechanistic studies showing an enhanced ability to attach to gestational tissues, induce stronger proinflammatory responses, and persist inside macrophages (Korir et al., 2014, 2017; Flaherty et al., 2019). Nonetheless, it is important to note that our clinical definitions of “invasive” vs. “colonizing” strain types may not be representative of each strain population. Although strains isolated from an active infection clearly demonstrate “invasive” potential, it is possible that strains designated as “colonizing” could also cause an infection in specific circumstances and host environments.

Although sialidases have no known role in GBS pathogenesis (Yamaguchi et al., 2016), these proteins were shown to be immunomodulatory in other bacterial species (Aruni et al., 2011; Sudhakara et al., 2019) while simultaneously promoting biofilm production and metabolism of host sugars (Hardy et al., 2017; Zaramela et al., 2019). The presence and abundance of sialidase was variable: the ST-1 and ST-17 MVs all contained sialidase, but the ST-12 MVs lacked it. In two prior studies of GBS MVs produced by a ST-7 strain, A909, neither C5a peptidase nor sialidase were identified (Surve et al., 2016; Armistead et al., 2021), further highlighting differences across strains. However, we cannot rule out the possibility that the abundance of these virulence factors was beneath the detection limit in those studies. Similarly, the previous analysis of GBS MVs highlighted the importance of hyaluronidase (Surve et al., 2016). This immunomodulatory factor has previously been shown to promote ascending infection, degrade host extracellular matrix components, and dampen the host immune response (Kolar et al., 2015). While we also found high levels of hyaluronidase in ST-17 MVs, our results further show that the ST-12 and ST-1 MVs contained significantly lower amounts of this protein. Additionally, the ST-1 strains lacked 124 proteins found in MVs from other lineages. Analysis of the ST-1 genomes detected the presence of the genes encoding 122 of these proteins, suggesting that lineage-specific composition is not due to genome divergence. Because we have previously shown that virulence gene expression in clinical isolates varies during infection of host cells (Korir et al., 2014), variable gene expression profiles could drive MV compositional differences. Alternatively, in the absence of varied gene expression, it is possible that there is strain-specific packaging of proteins within MVs. Further studies, however, are required to determine the mechanisms behind this altered composition.

It is also notable that multiple uncharacterized and hypothetical proteins were detected. Previous reports have demonstrated that in gram positive species, roughly 30–60% of all MV proteins map to the cytoplasm (Lee et al., 2009; Cao et al., 2020). While our results are consistent with this observation showing ∼22–52% of all proteins mapping to the cytoplasm, roughly 25–41% of the GBS MV proteins had an unidentifiable subcellular localization. Similar trends of ST-dependent enrichment of several hypothetical proteins were observed, with these representing some of the most highly abundant proteins. Although some uncharacterized proteins, such as those classified as putative ABC transporters, have predicted functions, their role in vesicle function or virulence is currently unknown. Future analyses must be undertaken to identify which proteins play a role in MV associated pathogenesis.

Through this study, we have also identified a shared proteome among MVs from phylogenetically distinct GBS strains. In total, 62 proteins were consistently found within GBS MVs regardless of the ST. Indeed, over 17% of these shared proteins were highly abundant, indicating that they may be important for MV functionality. Even though many of these proteins have yet to be characterized, we identified an abundance of transporter proteins in MVs suggesting a potential role in MV function. Some these shared proteins may be of value as potential MV markers in future studies.

While various mechanisms have been proposed for the biogenesis of gram positive MVs, those mechanisms important for GBS MV biogenesis are unclear (Brown et al., 2015; Briaud and Carroll, 2020). Our data demonstrate that diverse GBS strains produce MVs with consistent size distributions, indicating that GBS MV production is ubiquitous. Purported mechanisms of MV biogenesis in other pathogens include phage mediated biogenesis (Toyofuku et al., 2017, 2019), membrane budding during division (Vdovikova et al., 2017), and cell wall remodeling (Brown et al., 2015; Wang et al., 2018). Consistent with these mechanisms, our proteomics analysis revealed the presence of phage associated proteins, division septum-associated proteins and cell wall-modifying enzymes. Several of these proteins were also differentially abundant, with some proteins being more highly enriched in certain STs than others. For instance, phage proteins were enriched in ST-17 strains but were nearly absent in ST-12 and ST-1 strains. Although we observed similar enrichment of cell division proteins in ST-12 and ST-17 strains relative to the ST-1 strains, cell wall modifying proteins were most abundant in the ST-17 strains. Taken together, these data indicate that MVs are produced by diverse strains with varying traits; however, it is possible that the mechanisms for MV biogenesis are strain-dependent. Additional studies are needed to test this hypothesis.

Although our study has enhanced our understanding of the proteomic composition of GBS MVs, it has a few limitations. Because strains of each GBS lineage possess the same capsule (cps) type, it is difficult to differentiate between ST vs. cps effects. Another concern when dealing with MVs is the presence of non-vesicular contaminants. In some eukaryotic and prokaryotic systems where the composition of MVs is well defined, markers are used to assess purity (Sarker et al., 2014; Rompikuntal et al., 2015; Vanaja et al., 2016). Due to the relatively unknown composition of GBS MVs, however, we were unable to target specific markers to evaluate the purity. Rather, we relied on size exclusion chromatography followed by TEM to further remove non-vesicular proteins from each MV preparation. While some contaminant proteins are likely present, the purity of our preparations exceeds those performed in prior GBS studies (Surve et al., 2016; Armistead et al., 2021) and mimics protocols optimized for removing extravesicular macromolecules from Gram positive MVs (Surve et al., 2016; Dauros Singorenko et al., 2017; Mehanny et al., 2020; Armistead et al., 2021). Indeed, studies in *Staphylococcus aureus* and *Streptococcus mutans* have confirmed the presence of similar proportions of cytoplasmic and extracellular proteins within MVs (Lee et al., 2009; Cao et al., 2020). The MV isolation method used herein is standard for the field, however, it is important to note that the isolation is not complete, as a small proportion of MVs can remain associated with the bacterial surface post-isolation. Current protocols to isolate surface-associated MVs remain limited. Because our protocol was consistent across all production and proteomics experiments, the data could be directly compared across strains, thereby greatly enhancing our understanding of GBS MV composition across strains. Although other macromolecules have also been detected within GBS MVs (Surve et al., 2016), it is not clear whether these macromolecules have a ST-dependent composition and hence, further studies are warranted.

In summary, this analysis of GBS MVs from strains representing three phylogenetically distinct lineages demonstrates strain-dependent composition and production of MVs. Our data further show that MVs carry known virulence factors as well as proteins of unknown function that vary in abundance between strains, suggesting they may have an altered functionality or ability to promote virulence. Follow up studies elucidating virulence and immunomodulatory properties of GBS MVs isolated from a larger and more diverse strain collection are therefore warranted, particularly given the high level of variation in protein composition observed among only these six strains. Taken together, these findings further highlight the importance of strain variation in GBS pathogenesis and shed light on the potential role of MVs in virulence.

## Data Availability Statement

The datasets presented in this study can be found in online repositories. The names of the repository/repositories and accession number(s) can be found in the article/Supplementary Material.

## Author Contributions

CM, MGP, and SM designed the study. CM performed the laboratory work, conducted the analysis, and drafted the manuscript. MEP performed genome assembly and assisted with gene extraction analysis. MGP, SM, DA, and JG provided institutional support, guidance and resources. All authors contributed to and approved of the manuscript content.

## Conflict of Interest

The authors declare that the research was conducted in the absence of any commercial or financial relationships that could be construed as a potential conflict of interest.

## Publisher’s Note

All claims expressed in this article are solely those of the authors and do not necessarily represent those of their affiliated organizations, or those of the publisher, the editors and the reviewers. Any product that may be evaluated in this article, or claim that may be made by its manufacturer, is not guaranteed or endorsed by the publisher.

## Footnotes

1
https://github.com/nguyens7/tidyNano

2
https://db.psort.org

3
https://www.R-project.org
