# Supplementary material for: Production and Composition of Group B Streptococcal Membrane Vesicles Vary Across Diverse Lineages
Source: Front Microbiol. 2021 Nov 22;12:770499. doi: 10.3389/fmicb.2021.770499 (PMC8645895; doi:10.3389/fmicb.2021.770499)

Supplementary Figures

Figure S1: Growth of six group B streptococcal strains used for membrane vesicle (MV) isolation. Bacterial overnight cultures were back diluted 1:50 into fresh media and allowed to grow for 6 hours at 37°C with 5% CO_2. Optical Density $(OD)_{600}$ measurements were obtained hourly. Growth curves for each strain were performed in biological triplicate and analyzed and plotted using R. Hourly means were compared between strains using a Kruskal Wallis test followed by a Dunn's Test with a conservative Benjamini-Hochberg correction.

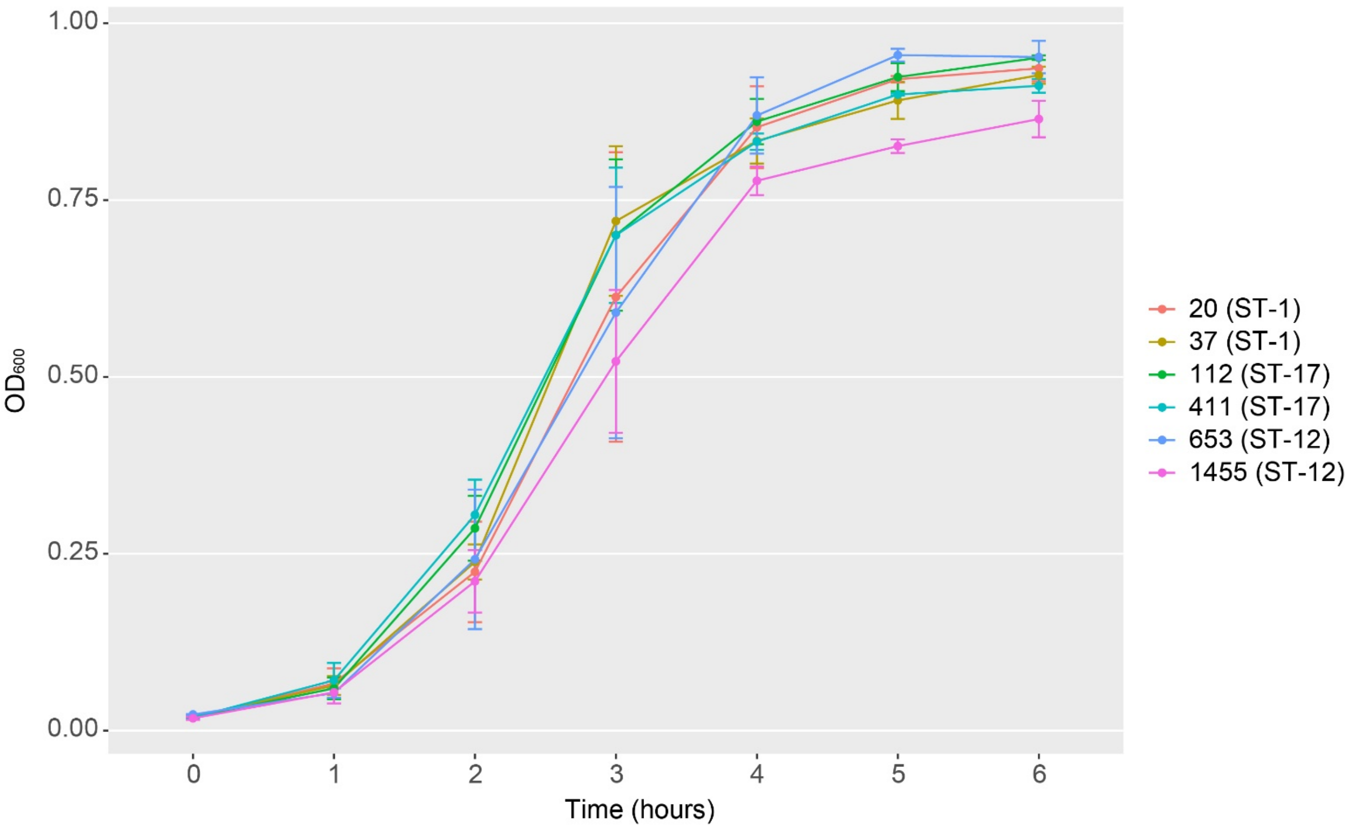

Figure S2: Membrane vesicle (MV) production varies by sequence type (ST). Vesicles were isolated using differential centrifugation and quantified by NanoSight analysis and plotted per bacterial colony forming units (CFUs). Data from each strain sharing the same ST were combined regardless of the clinical phenotype. Each colored dot represents a biological replicate. Black dots represent outliers that were identified by multiplying the interquartile range by 1.5, which was used to extend the upper and lower quartiles. When comparing production between ST-1 and ST-17, a two-sided students t-test was performed with significance defined as $p<0.05$.

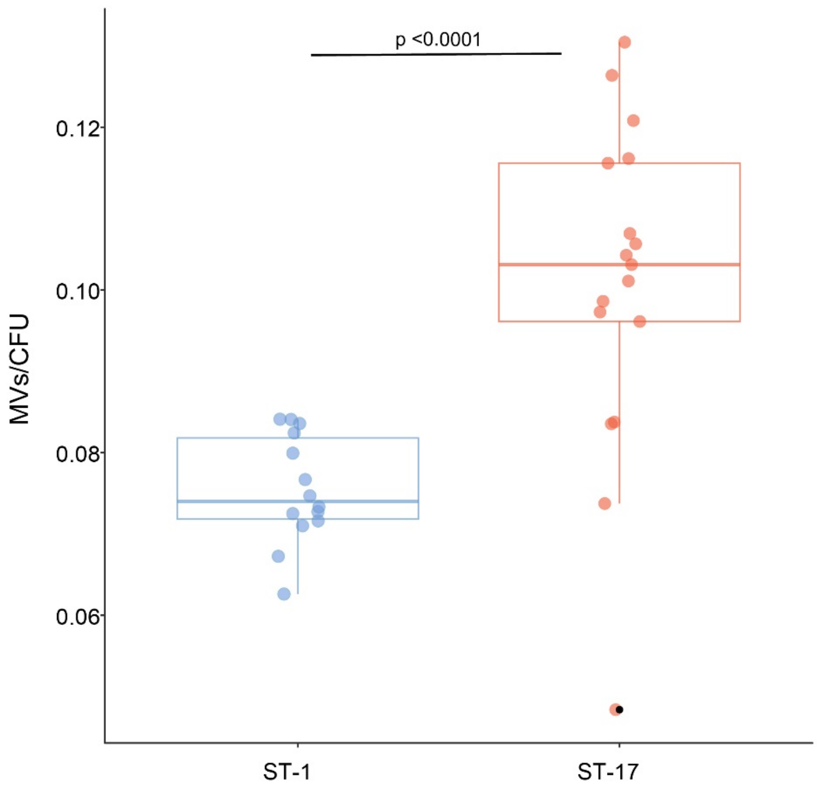

Figure S3: Principal component analysis (PCA) shows lack of association between clinical phenotype and membrane vesicle (MV) proteome composition. A PCA of the MV proteomes produced by six strains are shown after stratifying by clinical phenotype. The large central dot of each ellipse represents the mean point of the corresponding 95% confidence ellipse, while the smaller points represent individual proteomic samples. Axes percentages represent the amount of variation accounted for by the given principal component (PC).

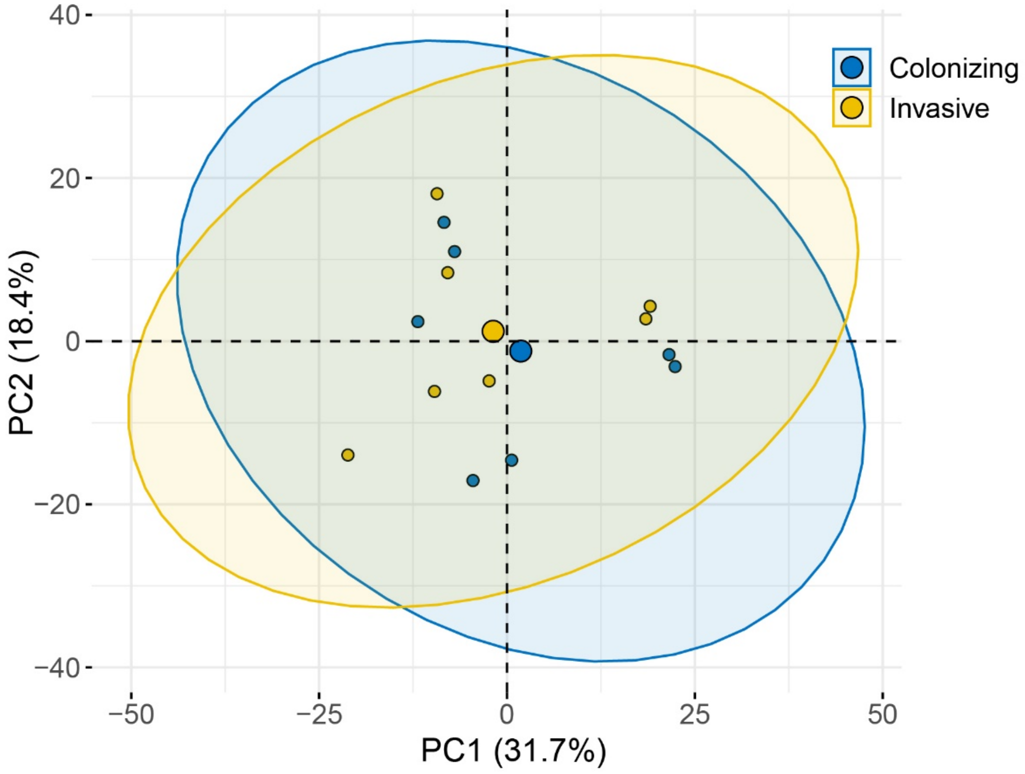

Figure S4: Differentially abundant membrane vesicle (MV) proteins associated with phages or unknown functions. The spectral counts of specific proteins were plotted after stratifying by the sequence type (ST). The median spectral count associated with each ST is represented within each box. The black dots represent a single biological replicate for a given strain. Statistical comparisons were performed using a Kruskal Wallis test. Multiple pairwise comparisons were made using the pairw.kw function in R, which uses a conservative Bonferroni correction method to correct for multiple hypothesis testing. Comparisons with p-values < 0.05 are denoted with an asterisk.

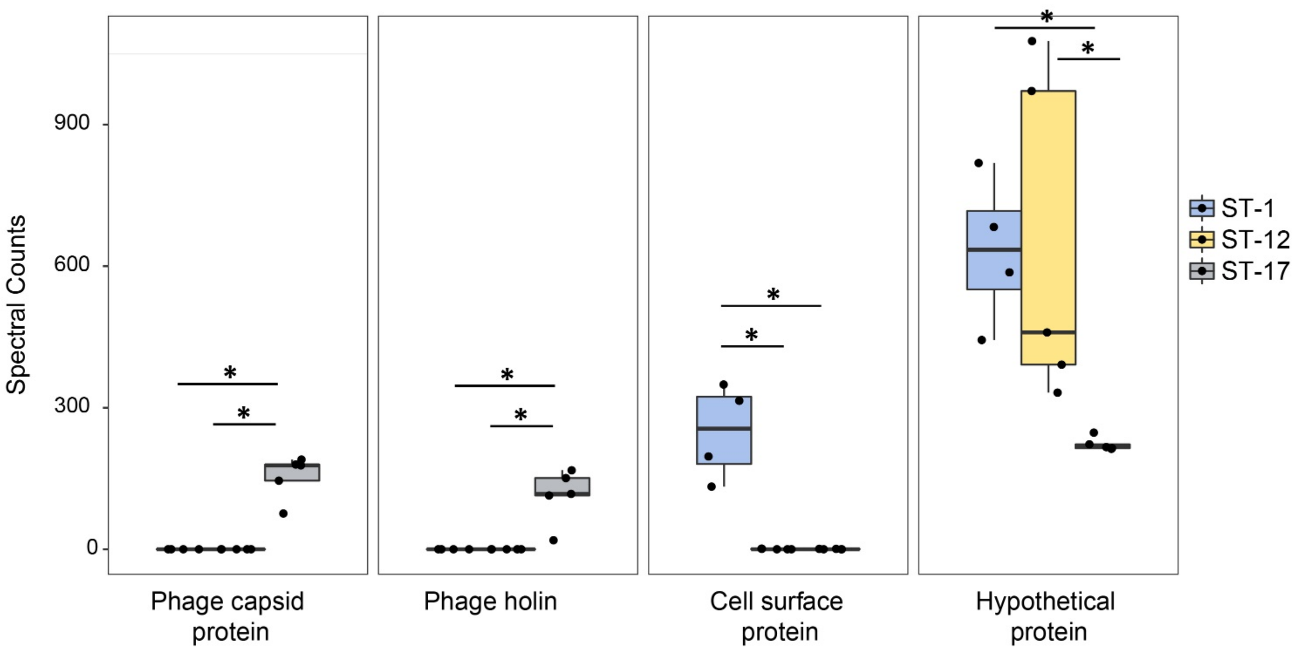

Figure S5: Membrane vesicle proteins associated with cellular division. The spectral counts of specific proteins linked to cellular division were plotted by sequence type (ST). The median spectral count associated with each ST is represented within each box. The black dots represent a single biological replicate for a given strain. Statistical comparison was performed using a Kruskal Wallis test. Multiple pairwise comparisons were made using the pairw.kw function in R, which uses a conservative Bonferroni correction method to correct for multiple hypothesis testing. Comparisons with p-values < 0.05 are denoted with an asterisk.

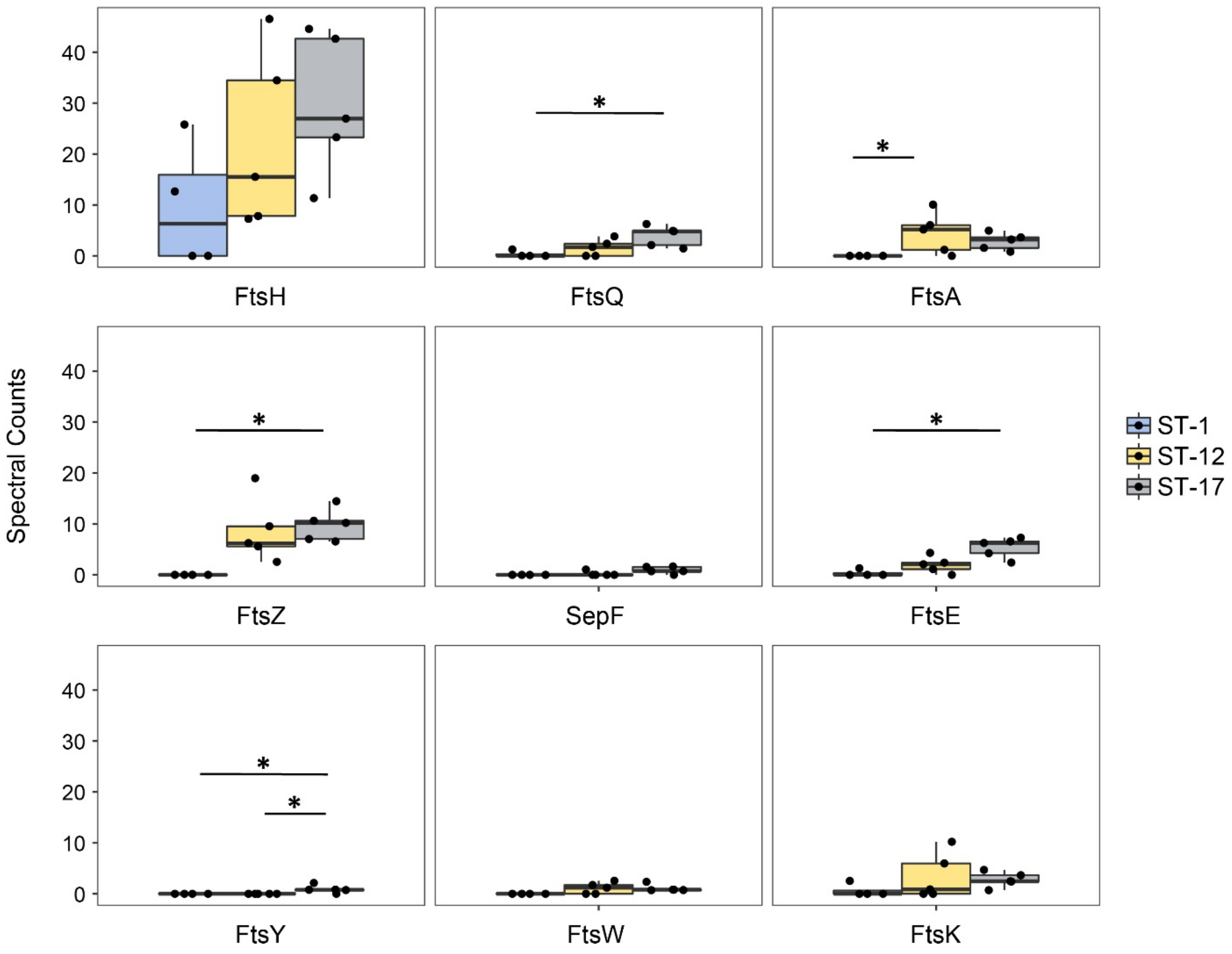

Figure S6: Membrane vesicle proteins associated with cell wall modification. The spectral counts of specific cell wall modification proteins were plotted by sequence type (ST). The median spectral count associated with each ST is represented within each box. The black dots represent a single biological replicate for a given strain. Statistical comparison was performed using a Kruskal Wallis test. Multiple pairwise comparisons were then made using the pairw.kw function in R, which uses a conservative Bonferroni correction method to correct for multiple hypothesis testing. Comparisons with p-values < 0.05 are denoted with an asterisk.

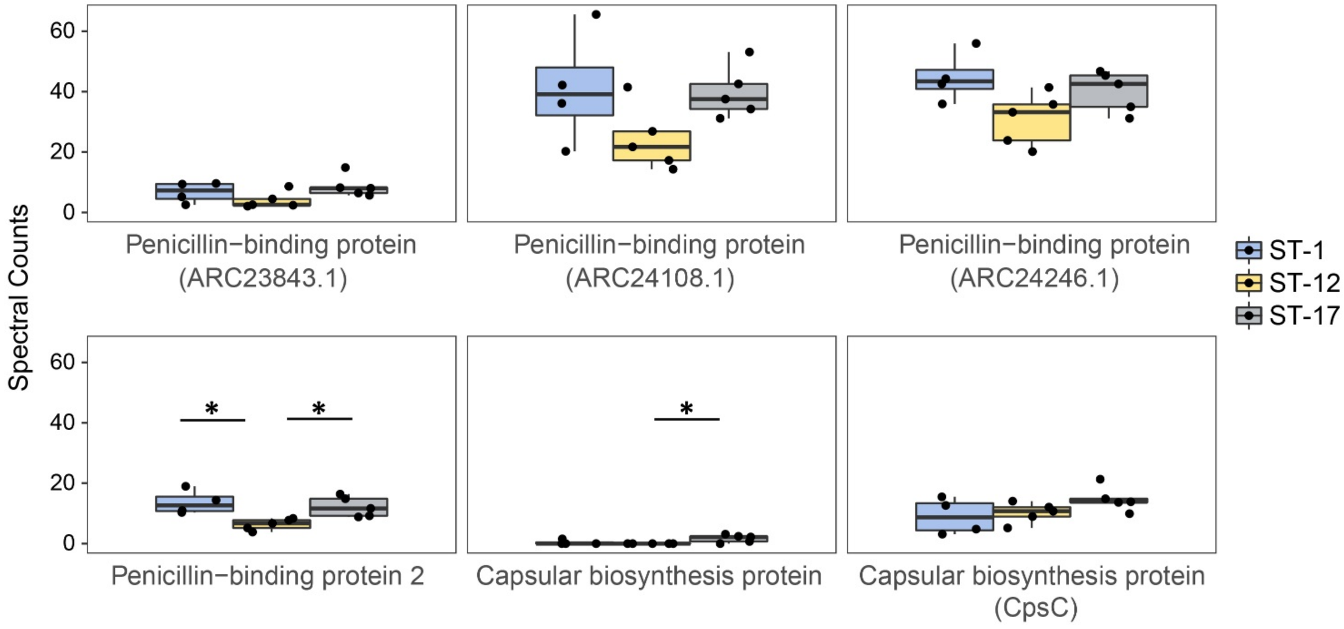

Supplement: Supplementary file 1 [file Data_Sheet_1.PDF]
